# A combination of recombinase polymerase amplification with CRISPR technology rapidly detects goose parvovirus with high accuracy and sensitivity

**DOI:** 10.3389/fcimb.2025.1566603

**Published:** 2025-06-16

**Authors:** Xiuqin Chen, Shizhong Zhang, Su Lin, Shao Wang, Meiqing Huang, Shaoying Chen, Shilong Chen

**Affiliations:** Institute of Animal Husbandry and Veterinary Medicine, Fujian Academy of Agricultural Science, Fuzhou, Fujian, China

**Keywords:** goose parvovirus, CRISPR/Cas12a, recombinase polymerase amplification, nucleic acid, portable

## Abstract

**Background:**

Goose parvovirus (GPV) poses a significant threat to the waterfowl industry, necessitating reliable detection methods. However, conventional techniques are often time-consuming, equipment-dependent, or lack sufficient sensitivity for detecting early-stage infection. In contrast, emerging CRISPR/Cas12a-based systems offer a promising alternative for rapid, sensitive, and on-site diagnostics.

**Methods:**

We developed and optimized a recombinase polymerase amplification (RPA)-CRISPR/Cas12a assay targeting the conserved *VP3* gene of GPV. The analytical and diagnostic performance of this assay was rigorously validated using plasmid standards and clinical specimens from both experimentally infected and field-collected ducklings.

**Results:**

Our developed assay combines RPA with CRISPR/Cas12a technology for rapid GPV nucleic acids detection. This method achieves a detection limit of 10 copies/*μ*L of the *VP3* gene within one hour, demonstrating high sensitivity and rapid turnaround. The assay exhibited exceptional specificity, with no cross-reactivity against other waterfowl viruses, and showed robust reproducibility, with intra- and inter-assay coefficients of variation consistently below 5.0%. Clinical validation using 42 field samples confirmed a diagnostic sensitivity of 100% and 95.5% specificity, showing superior performance to real-time quantitative PCR (qPCR) in both metrics. Furthermore, the assay supports flexible visual readouts using portable blue light transilluminators, facilitating on-site interpretation.

**Conclusions:**

This study established a highly field-deployable RPA-CRISPR/Cas12a assay for rapid, visual detection of GPV with outstanding sensitivity and specificity. Its capability for instrument-free on-site diagnosis via blue light transillumination makes this approach particularly promising for resource-limited settings.

## Introduction

Waterfowl parvoviruses belong to the genus *Dependovirus* within the family Parvoviridae. They are categorized into two primary groups based on host susceptibility and complete genomic characteristics (Zádori et al., 1995). These groups comprise Muscovy duck parvovirus (MDPV) and goose parvovirus (GPV). MDPV specifically affects Muscovy ducklings (Cheng et al., 1993), while GPV is responsible for Derzy’s disease in both goslings (Derzsy, 1967) and Muscovy ducklings (Cheng et al., 2008; Wang et al., 2015, 2013), as well as short beak and dwarfism syndrome (SBDS) in mule ducks and Cherry Valley ducks (Chen et al., 2016; Xiao et al., 2017). Muscovy duck-origin GPV (MDGPV) arises from natural recombination between MDPV and GPV (Liu et al., 2024; Wang et al., 2015), showing a diagnostic feature of intestinal embolism similar to that observed in Derzy’s disease (Wang et al., 2020, 2019). MDPV, classical and recombinant GPV infections, can cause high morbidity and mortality in domestic waterfowl (Cheng et al., 1993; Glavits et al., 2005). Additionally, the short beak and dwarfism syndrome virus (SBDSV), a distinct GPV originating from ducks (Chen et al., 2016; Wang et al., 2016), presents a lower morbidity rate (2% to 10%) (Chen et al., 2015), yet infected ducks exhibit an average weight reduction of approximately 1 kg at slaughter compared to healthy counterparts (Chen et al., 2015). These infections have posed significant economic challenges in domestic waterfowl production across Asia, Europe, and North America (Chen et al., 2016; Cheng et al., 2008; Gough et al., 2005; Jansson et al., 2007; Poonia et al., 2006; Woolcock et al., 2000).

Traditional detection methods for GPV include virus isolation (Chen et al., 2016), serological assays such as ELISA, latex agglutination, and indirect immunofluorescence assays (IFA) (Chen et al., 2016; Takehara et al., 1999; Zhang et al., 2020), and nucleic acid-based amplification techniques (Lin et al., 2019; Luo et al., 2018). While virus isolation remains the gold standard for diagnosing GPV infection. Nevertheless, given their labor-intensive and time-consuming nature, as well as the need for highly skilled personnel, these techniques fall short in meeting the urgent demand for rapid field diagnostics. Serological tests, though rapid and user-friendly, are limited by their inability to detect the virus in early infection stages and exhibit low sensitivity (Luo et al., 2018). Molecular advancements have introduced conventional PCR and real-time quantitative PCR (qPCR), offering high sensitivity and specificity (Lin et al., 2019; Luo et al., 2018; Wozniakowski et al., 2010). However, these methods are hampered by long turnaround times and the necessity for sophisticated equipment, which restricts their applicability in resource-limited settings. Isothermal amplification techniques, such as recombinase polymerase isothermal amplification (RPA), have emerged as promising alternatives by overcoming some of these limitations (Daher et al., 2016). Nonetheless, the application of RPA in on-site detection has been impeded by the risk of false-positive results due to aerosol contamination (Hu et al., 2022).

The recent advent of the CRISPR/Cas system, particularly the trans-cleavage activity mechanism, has revolutionized nucleic acid detection (Chen et al., 2018; Gootenberg et al., 2017; Li et al., 2018). Cas12a (Cpf1), for instance, can specifically recognize and bind to target double-stranded DNA (dsDNA) or single-stranded DNA (ssDNA) with a protospacer adjacent motif (PAM) sequence, facilitated by guide RNAs (gRNAs) (Chen et al., 2018). This binding leads to the cleavage of surrounding ssDNA, resulting in the release of fluorophores and quenching groups, thereby transforming target sequence information into a detectable fluorescent signal. The integration of RPA pre-amplification with this system has led to the development of the first CRISPR-Dx platform, DETECTR, which achieves attomolar sensitivity for DNA detection (Chen et al., 2018). Unlike PCR, which requires precise thermal cycling, CRISPR-based detection operates effectively at physiological or room temperatures, making it highly adaptable for on-site use (Gootenberg et al., 2017; Hu et al., 2023; Wei et al., 2022; Wu et al., 2022). However, the application of the CRISPR/Cas12a system combined with RPA for GPV detection remains unexplored.

**Figure 1:**
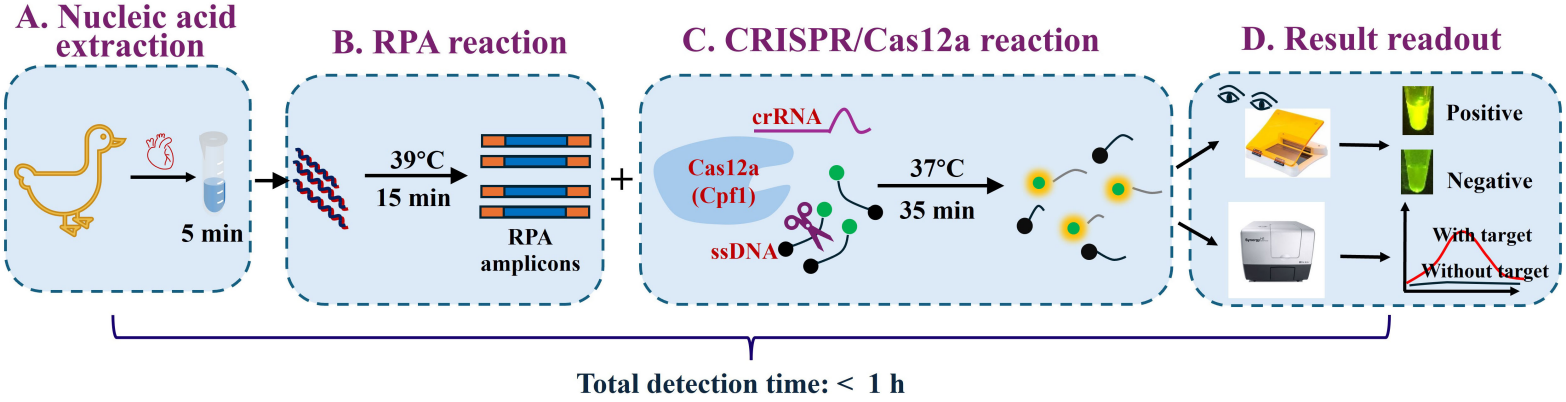
Schematic of the RPA-CRISPR/Cas12a assay for GPV detection. The workflow comprises four main steps: **(A)** Cardiac tissue is lysed in nucleic acid lysis buffer for 5 min. **(B)** The resultant lysate is directly subjected to recombinase polymerase amplification (RPA) at 39°C for 15 min to amplify the target sequence. **(C)** The CRISPR RNA (crRNA)-guided Cas12a ribonucleoprotein (RNP) specifically binds to the double-stranded DNA (dsDNA) amplicons. Formation of the RNP/dsDNA complex activates the trans-cleavage activity of Cas12a, which subsequently cleaves single-stranded DNA (ssDNA) reporter molecules, producing a fluorescent signal. **(D)** Fluorescence readouts can be visualized by the naked eye under blue light (470 nm excitation) using a portable blue light transilluminator or quantified using a multimode microplate reader.

This study introduces a novel CRISPR/Cas12a-based assay targeting the *VP3* gene of GPV. The assay delivers rapid and accurate results within a 1-hour turnaround time, significantly expediting the diagnostic process. Moreover, the use of a portable blue light transilluminator enables visual detection without the need for sophisticated equipment, rendering the assay particularly advantageous for resource-limited settings (**Figure 1**). By providing a reliable and user-friendly diagnostic tool, this study paves the way for efficient surveillance and control of GPV in domestic waterfowl populations.

## Materials and methods

### Reagents

NEBuffer 2.1, NEBuffer 3.1, CutSmart buffer, and EnGen Lba Cas12a protein were obtained from New England Biolabs (Beijing, China). 10 × Cas12a reaction buffer was purchased from Guangzhou Magigen Biotechnology Co., Ltd. (Guangzhou, China). The nucleic acid rapid lysis solution was purchased from Shanghai Kanglang Biotechnology Co., Ltd. (Shanghai, China). The TwistAmp^®^ Basic kit was purchased from TwistDx Ltd. (Cambridge, UK). PerfectStart^®^ Green qPCR SuperMix and *EasyPure* ^®^ Viral DNA/RNA Kit were acquired from TransGen Biotechnology Co., Ltd. (Beijing, China).

### Animals and viruses

A total of thirty-six 23-day-old healthy Muscovy ducklings were examined for waterfowl parvovirus antibodies using the previously described latex agglutination inhibition assay (LAI) (Chen et al., 2016).

The viruses used in this study include the following: SBDSV, MDGPV, classical GPV (cGPV), MDPV, Muscovy duck reovirus (MDRV), novel duck reovirus (NDRV), duck plague virus (DPV) and duck adenovirus B2 (DAdV B2). They were identified and preserved at the Institute of Animal Husbandry and Veterinary Medicine, Fujian Academy of Agricultural Sciences.

### Design of RPA primers and CRISPR RNA

The complete genome nucleotide sequences of GPV were retrieved from the National Center for Biotechnology Information (NCBI) database (http://www.ncbi.nlm.nih.gov/). The identification of conserved regions was carried out through multiple sequence alignments using the MegAlign module in the DNASTAR software (Version 7.1). This analytical approach conclusively identified the *VP3* gene as the most conserved genomic region of GPV, a finding fully consistent with prior reports (Ma et al., 2022; Dong et al., 2019; Yang et al., 2017). Three primer sets targeting the *VP3* gene were generated using Primer Premier version 5.0. software (PREMIER Biosoft, CA, USA) in accordance with the TwistAmp assay design manual, and both hairpin and cross dimer were assessed. The CRISPR RNA (crRNA) was designed to target the *VP3* gene of GPV employing the online tool Benchling (https://www.benchling.com/crispr/). NCBI-BLAST was used to conduct the specificity analysis of RPA primers. The sequence conservative analysis of crRNAs was performed using the ClustalW program (https://www.genome.jp/tools-bin/clustalw). The oligonucleotides utilized in this research were synthesized by Sangon Biotech (Shanghai, China). Detailed information regarding the designed primers and crRNA are shown in **Table 1**.

**Table 1:** Sequences of oligonucleotides utilized in this study.

Name	Sequence (5’to 3’)	Product length	Ref.
RPA-F1	CAAGATCTTCAATGTTCAAGTCAAGGAAGTC	259 bp	This study
RPA-R1	CAATGACCGTAGCGCATTCTACTGCTTAGAG		
RPA-F2	CAAGGAAGTCACAACGCAGGATCAGACAAAG	200 bp	
RPA-R2	TACTGCACAATGCACACCAACCAGAATGGAG		
RPA-F3	CATTGCAAACAATCTCACCTCAACAATCCAAG	119 bp	
RPA-R3	GACCATGCCGCCGTTCCCGTCGGATGTCTATG		
qPCR-F	GAGGTAGACAGCAACAGAAA	343 bp	Lin et al., 2019
qPCR-R	GCTCGTCCGTGACCATA		
crRNA*	UAAUUUCUACUAAGUGUAGAUCGGAUGAUGAGCACCAACUC		This study
FQ reporter	FAM-TTATT-BHQ		This study

*The spacer sequences of the crRNA, which are responsible for recognizing and binding to the target, are marked with an underline.

### Establishment of RPA assay

RPA was performed following the manufacturer’s protocol for pre-amplifying DNA sequences. Briefly, a 50 *μ*L RPA mixture was prepared, consisting of 29.5 *μ*L of rehydration buffer, 480 nM of each primer, 1 *μ*L of target template, and 12.2 *μ*L of sterile nuclease-free water, along with 2.5 *μ*L of 280 nM magnesium acetate. A negative control was included by replacing the DNA template with sterile nuclease-free water. The RPA was conducted at 39°C in a water bath for 15 min, and subsequently incubated at 95°C for 5 min. Purification of the RPA products was carried out using a Universal DNA Purification Kit (TianGen, Beijing, China). Evaluation of the reaction products was conducted through gel electrophoresis. Primer pairs exhibiting superior performance were selected through gel electrophoresis screening.

### CRISPR/Cas12a-mediaed nucleic acid detection and visualization

The LbaCas12a reaction was conducted in a total volume of 30 *μ*L. Firstly, 100 nM Cas12a was pre-incubated with 200 nM crRNA in 10 × Magigen reaction buffer at 37°C for 5 min to form the ribonucleoprotein (RNP) complex. Subsequently, 200 nM ssDNA reporter and 1 *μ*L of RPA amplicons were added to the RNP complex, then the solution was immediately transferred to a 384-well black polystyrene microplate (Costar). Real-time fluorescence analysis was carried out at 37°C using the multimode microplate reader Bio Tek Synergy H1, with fluorescence measurements recorded every 1 min for 30 min. The excitation and emission wavelengths were set at 485 and 525 nm, respectively. To obtain the visual detection results of the samples, the tubes were placed on a portable blue-light instrument (TianGen, Beijing, China). The visual detection results were photographed using a smartphone in a dark environment.

### Evaluation of the limit of detection and specificity of the RPA-CRISPR/Cas12a assay

To assess the LOD of the RPA-CRISPR/Cas12a assay, 1 *μ*L of 10-fold serially diluted plasmid DNA ranging from 10^0^ to 10^3^ copies/*μ*L was used as templates for the RPA reaction. Subsequently, 1 *μ*L of the positive RPA amplicons were used to trigger the CRISPR/Cas12a cleavage system. Each reaction was conducted in triplicate, with the negative RPA amplicons serving as the negative control. In terms of specificity evaluation, the DNA of five waterfowl-origin viruses, including MDPV, MDRV, NDRV, DPV, and DAdV B2, were tested.

### Real-time qPCR assay

The qPCR assay (Lin et al., 2019) for GPV detection was performed following the instructions of the Roche LightCycler^®^ 96 instrument (Roche Diagnostics, Germany). Briefly, the qPCR reaction mixtures consisted of 10 *μ*L of PerfectStart^®^ Green qPCR SuperMix, 0.5 *μ*L of each forward and reverse primer (10 *μ*M), 1 *μ*L of DNA template, and 8 μL of nuclease-free water to a final volume of 20 *μ*L. The qPCR amplification conditions were as follows: pre-denaturation at 95°C for 5 min, followed by 40 cycles of denaturation at 95°C for 15 s, annealing at 60°C for 10 s, and extension at 72°C for 15 s. Samples with a cycle threshold (Ct) value < 30 and a melting temperature (Tm) of 86.15 ± 0.26°C, exhibiting a distinct single peak, were considered positive for GPV.

### Repeatability and reproducibility analysis of RPA-CRISPR/Cas12a assay

Three different concentrations of standard plasmid (10^6–^10^4^ copies/*μ*L) were tested for the intrabatch and interbatch assays. For repeatability (intra-assay) analysis, three different concentrations of standard plasmid were tested in three independent runs. Reproducibility (inter-assay) was determined in three independent runs conducted by different individuals on different days, with results analyzed using the coefficient of variation (CV).

### Validation of the RPA-CRISPR/Cas12a assay using mock/actual samples

All experimental procedures were reviewed and approved by the Institute of Animal Husbandry and Veterinary Medicine, Fujian Academy of Agricultural Science Animal Care and Use Committee (license number: MYLLSC2024-007).

To assess the practical application value of the RPA-CRISPR/Cas12a assay, thirty-six 23-d-old Muscovy ducklings were randomly allocated into two groups, each consisting of 18 ducklings. Ducklings in Group 1 received an intramuscular injection in the leg with 0.5 ml of MDGPV strain JS, a 5th-passage allantoic fluid virus with a titer of 2^10.0^ LA, serving as the positive control. Conversely, ducklings in Group 2 were administered 0.5 ml of sterile phosphate-buffered saline (PBS) as a mock control. All Muscovy ducklings were housed in isolators, and their clinical signs were meticulously monitored daily for a total of 14 days. Following the challenge, serum, oropharyngeal swabs, cloacal swabs, and tissue samples from the heart, lung, spleen, liver, kidney, and pancreas samples were collected from the mock-injected ducklings at 2, 5, 10, and 14 days post infection (dpi). Additionally, 42 field-isolated samples were collected from ducklings suspected of GPV infection in Tutian or Zhangzhou, Fujian Province. Nucleic acids were extracted from heart tissue samples using a rapid lysis solution, with 1 μL of the extracted nucleic acids serving as the template for both the RPA-CRISPR/Cas12a method and the qPCR assay. The results obtained were confirmed using an IFA, as previously described (Chen et al., 2016).

### Statistical analysis

The statistical analysis was performed using GraphPad Prism 8.0 (GraphPad, USA). The unpaired Student’s t-test was used to analyze differences between two groups. ^∗∗∗∗^ represents *P* < 0.0001, ^∗∗^ represents *P* < 0.01, and ns represents *P* > 0.05. The data were presented as mean ± SD, derived from three independent experiments. LOD, specificity, positive predictive value (PPV), and negative predictive value (NPV) were calculated using established statistical formulas. Exact 95% confidence intervals (CI) for these performance metrics were determined using the Clopper-Pearson method, which employs binomial probability calculations to ensure conservative interval estimates.

## Results

### Verification of the RPA-CRISPR/Cas12a assay for GPV detection

**Figure 2:**
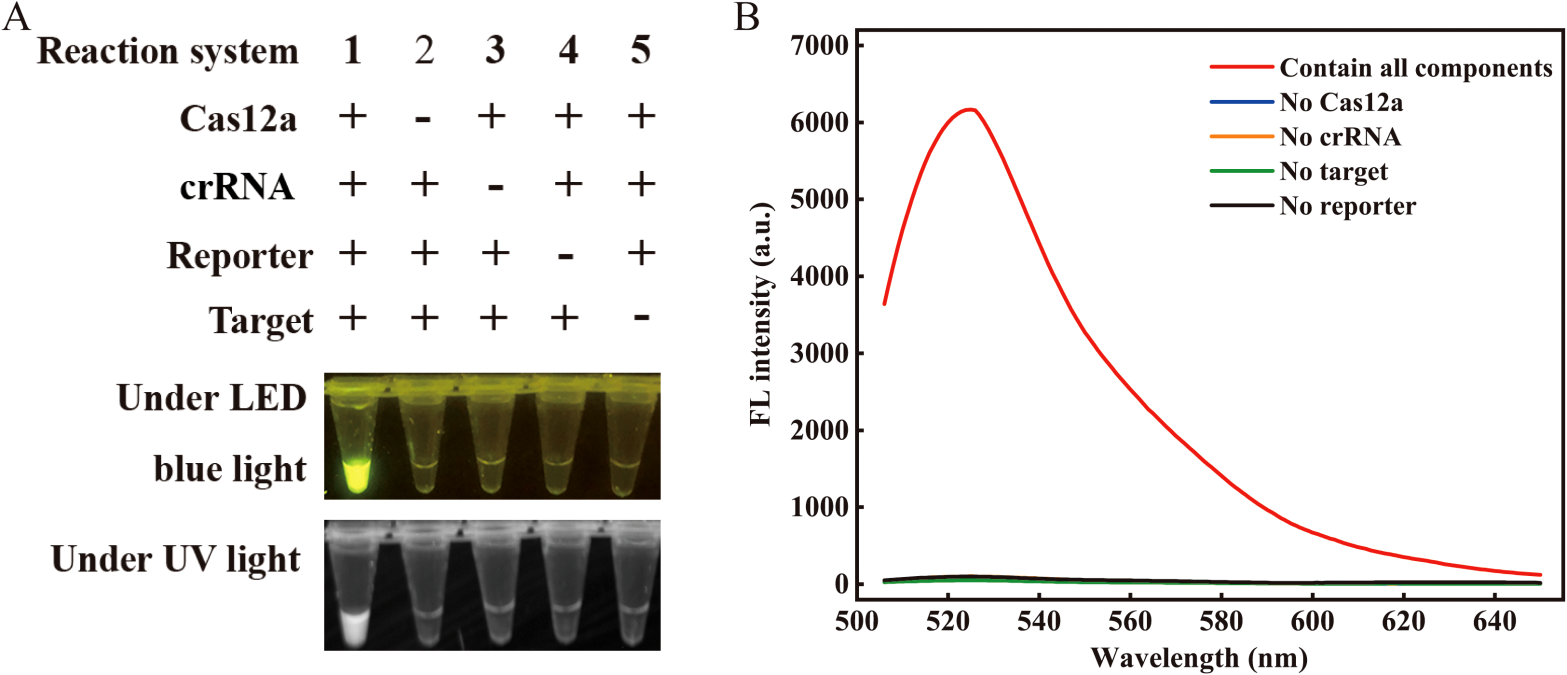
Feasibility validation of the RPA-CRISPR/Cas12a assay. **(A)** Validation of the Cas12a cleavage activity under blue light (470 nm) and UV light. The “+” refers to the presence of a reaction ingredient, while “-” represents their absence. **(B)** Spectrogram fluorescence of the RPA-CRISPR/Cas12a assay. Apart from the red curve, all other curves depict reactions where a particular single component was excluded while retaining all other components and enzymes (λex 485 nm, λem 525 nm). Target denotes the target nucleic acid sequence. The concentrations of Cas12a, crRNA, and ssDNA reporter were 100 nM, 200 nM, and 200 nM, respectively.

To examine the feasibility of the proposed assay, we prepared five reaction systems (reactions #1–5; **Figure 2**). A plasmid containing a 259 bp fragment of the *VP3* gene was served as the target. After a 20-minute incubation at 37°C, only reaction #1, comprising all components, showed a strong fluorescence signal when illuminated with LED blue or UV light. Conversely, the absence of certain components resulted in no detectable fluorescence signals (**Figure 2A**). The feasibility of the visual readout was further confirmed by measuring the fluorescent intensity (**Figure 2B**). These results demonstrated the feasibility of using the RPA-CRISPR/Cas12a assay for detecting GPV.

### Screening of the optimal RPA primer pairs

Three pairs of RPA primers were designed to target the *VP3* gene of GPV. The optimal RPA primers were validated through agarose gel electrophoresis. As depicted in **Supplementary Figure S1**, the 259 bp RPA product corresponding to the first pair of primers had the brightest and clearest band, with no nonspecific bands present. Consequently, the first pair of primers was selected for further experimentation. Moreover, the Sanger sequencing result further demonstrated the efficacy of the RPA primers (**Supplementary Figure S2**).

### Evaluation of the optimized conditions

**Figure 3:**
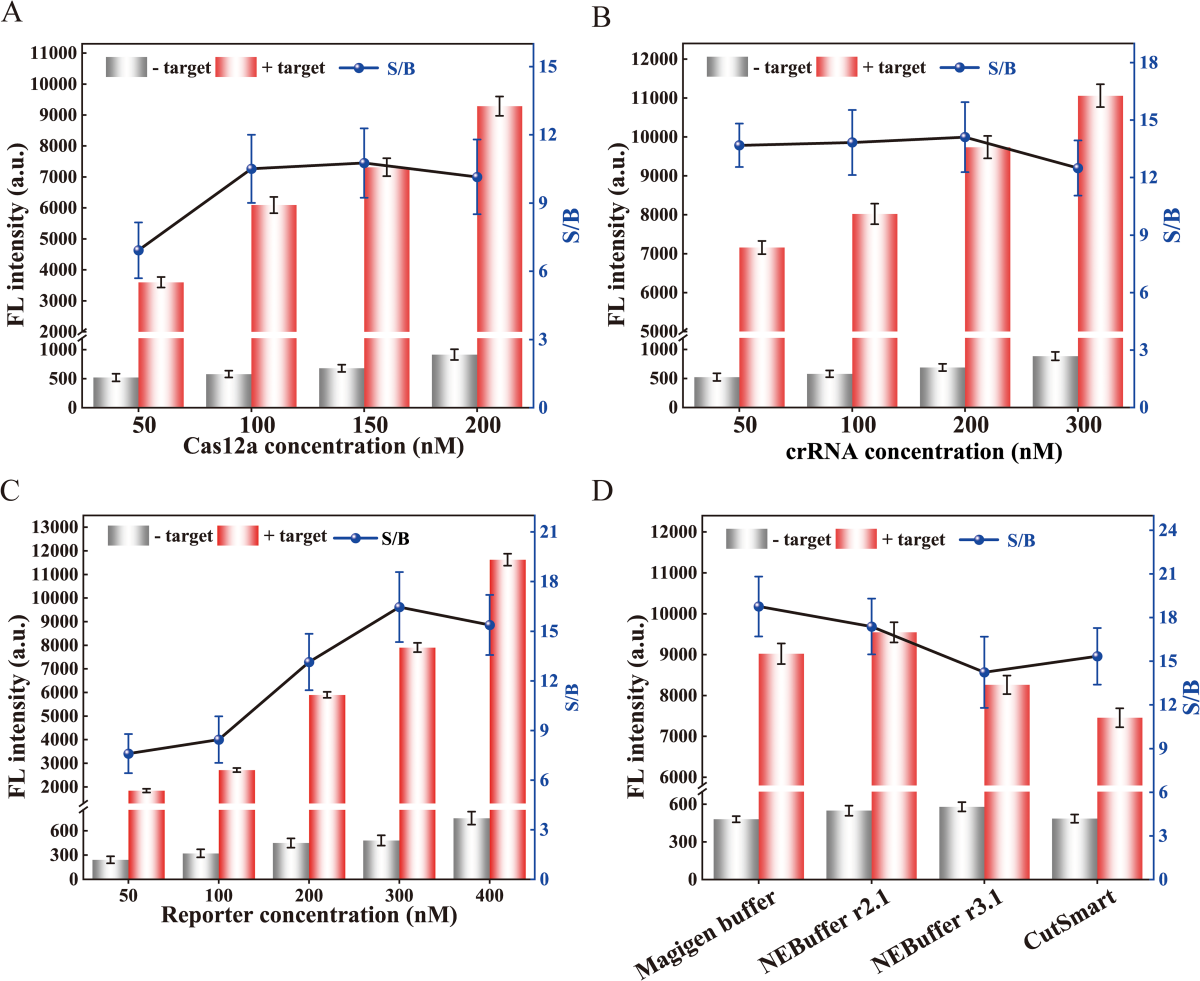
Optimization of reaction conditions for the RPA-CRISPR/Cas12a assay. Optimization of **(A)** Cas12a concentration, **(B)** crRNA concentration, and **(C)** reporter concentration. **(D)** Effect of different buffers on *trans*-cleavage efficiency of CRISPR/Cas12a. The signal-to-background ratio was determined based on the ratio of positive to negative fluorescence intensity. The fluorescence intensity at 30 min of the reaction was displayed. Error bars represent the mean of three replicates ± standard deviation (SD).

Several parameters were optimized to enhance the analytical performance, including the concentration of Cas12a, crRNA, ssDNA reporter, and buffers. The concentration of Cas12a was found to have a significant impact on the *trans*-cleavage efficiency of the CRISPR/Cas12a. Therefore, various concentrations of Cas12a enzyme were first optimized. As shown in **Figure 3A**, the fluorescence intensity was highest at 200 nM Cas12a. However, the signal-to-background ratio (S/B) was lower than that at 150 nM. Therefore, the optimal concentration of Cas12a was 150 nM. Furthermore, the concentration of crRNA plays a pivotal role in determining the optimal signal readout. Therefore, the concentration of crRNA was also optimized. The optimal concentration, which yielded the highest S/B ratio, was 200 nM (**Figure 3B**). For the ssDNA reporter, 300 nM was the optimal concentration as it provided the highest S/B ratio despite an increase in fluorescence intensity with higher concentrations (**Figure 3C**). Additionally, the chemical environment of the Cas12a may impact its *trans*-cleavage efficiency. Therefore, we selected four commercial reaction buffers to investigate. We found that the Magigen buffer gave the highest S/B ratio (**Figure 3D**), therefore it was chosen for subsequent analyses.

### LOD and specificity of the RPA-CRISPR/Cas12a assay

**Figure 4:**
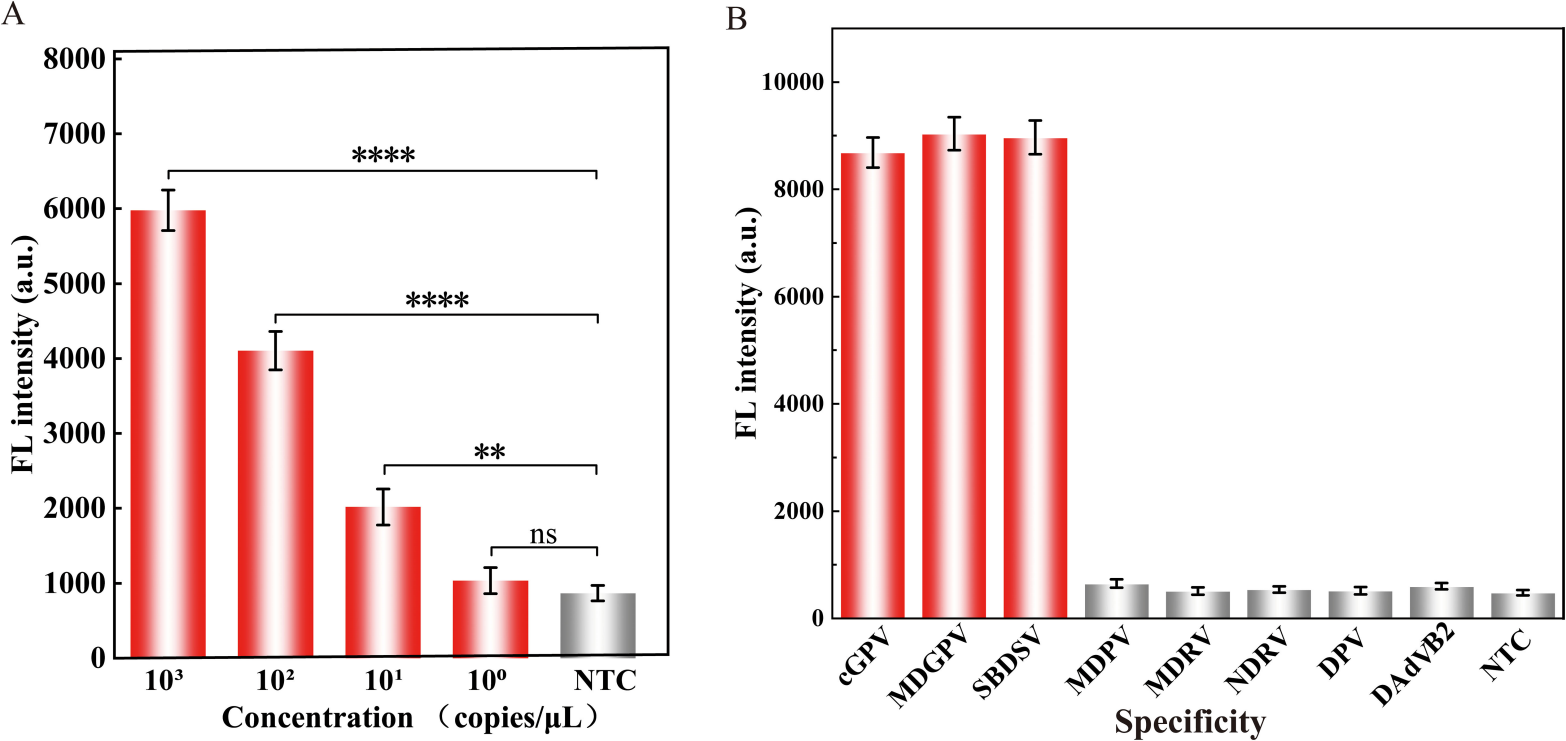
Properties evaluation of the RPA-CRISPR/Cas12a assay for detecting GPV. The **(A)** sensitivity and **(B)** specificity analysis of the proposed assay. Equal concentrations of the nucleic acids of the virus were used in all analyses. The fluorescence intensity at 30 min of the reaction was displayed. Error bars represent the mean of three replicates ± standard deviation (SD). NTC implies the absence of template control. ∗∗∗∗ represents *P* < 0.0001, ∗∗ represents *P* < 0.01, and ns represents *P* > 0.05.

To assess the LOD of the Cas12a-mediated cleavage assay, 10-fold serial dilutions of the standard plasmid pMD-19T-*VP3* ranging from 10^0^ to 10^3^ copies/*μ*L were used as the template. As shown in **Figure 4A**, the assay successfully detected plasmids containing as low as 10 copies/*μ*L of the *VP3* gene.

The specificity of the assay was evaluated using viruses that commonly infect ducks, including MDPV, MDRV, NDRV, DPV, and DAdV B2. As illustrated in **Figure 4B**, the fluorescence intensity of cGPV, MDGPV, and SBDSV were significantly higher than those of the other duck viruses, confirming that the assay had high specificity without cross reactions with non-GPV targets.

### Analytical the repeatability and reproducibility of the RPA-CRISPR/Cas12a assay

To further validate the performance of the established assay, we conducted experiments to assess its repeatability and reproducibility. CVs were calculated for three different concentrations of standard plasmid in both intra-batch and inter-batch analyses. The results indicated that the constructed assay possesses excellent reproducibility and repeatability in detecting GPV, with an intra-assay CV for fluorescence intensity ranging from 1.54% to 2.46%, and an inter-assay CV ranging from 2.41% to 3.18% (**Table 2**).

**Table 2:** Results of repeatability and reproducibility analysis of the RPA-CRISPR/Cas12a assay.

Concentration of standard plasmid (copies/*μ*L)	Repeatability (intra-bath assay)		Reproducibility (inter-bath assay)	
	mean ± SD	CV (%)	mean ± SD	CV (%)
10^6^	9588.33 ± 147.79	1.54	9798.67 ± 210.09	2.14
10^5^	8620.00 ± 165.31	1.92	8683.33 ± 252.94	2.91
10^4^	7426.00 ± 182.95	2.46	7600.00 ± 242.03	3.18

Mean, the average of fluorescence intensity of three independent RPA-CRISPR/Cas12a runs.

SD, standard deviation; CV, coefficient of variation.

### Application of the assay on clinical samples

First, we applied the proposed strategy on multiple mock samples to evaluate its practical applicability. As shown in **Table 3**, the RPA-CRISPR/Cas12a assay successfully detected GPV-DNA in all tissue, blood, oropharyngeal swabs, and cloacal swabs samples at both 2 and 5 dpi. Furthermore, this assay maintained its sensitivity, detecting GPV-DNA in blood and cloacal swab samples even at 10 dpi. However, at 15 dpi, GPV-DNA was undetectable in any of the mock samples. In contrast, as shown in **Table 4**, the qPCR assay was only capable of detecting GPV-DNA in blood samples at 2 dpi. These results demonstrate that the RPA-CRISPR/Cas12a assay exhibits superior sensitivity than the qPCR assay for detecting GPV-DNA in clinical samples.

**Table 3:** Detection of GPV-DNA in mock samples using the RPA-CRISPR/Cas12a assay.

Days post infection	Blood	Heart	Liver	Spleen	Lung	Kidney	Pancreas	Oropharynx	Cloacal
2	+	+	+	+	+	+	+	+	+
5	+	+	+	+	+	+	+	+	+
10	+	–	–	–	–	–	–	–	+
15	–	–	–	–	–	–	–	–	–

+, positive; −, negative.

**Table 4:** Detection of GPV-DNA in mock samples using the qPCR assay.

Days post infection	Blood	Heart	Liver	Spleen	Lung	Kidney	Pancreas	Oropharynx	Cloacal
2	+	–	–	–	–	–	–	–	–
5	–	–	–	–	–	–	–	–	–
10	–	–	–	–	–	–	–	–	–
15	–	–	–	–	–	–	–	–	–

+, positive; −, negative.

**Figure 5:**
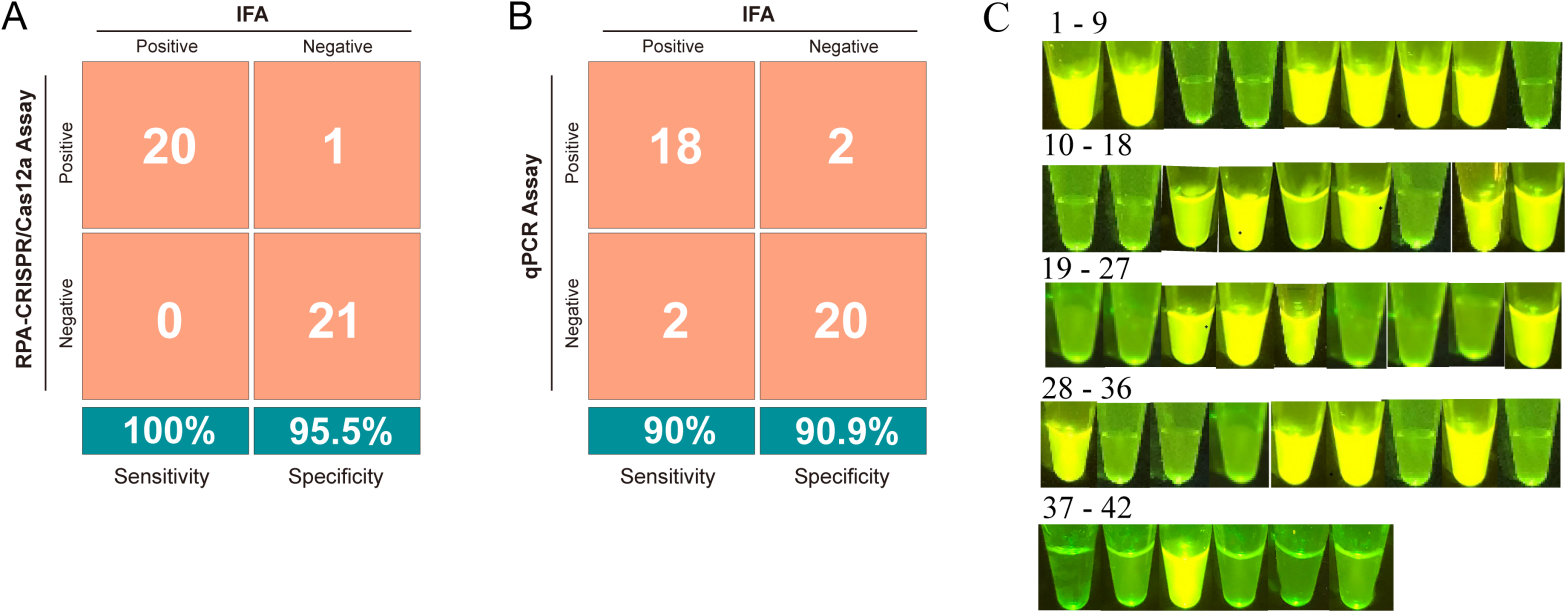
Evaluation of the RPA-CRISPR/Cas12a assay for detecting GPV in clinical samples. **(A)** Confusion matrix illustrating the comparison of results between the RPA-CRISPR/Cas12a assay and indirect immunofluorescence assay (IFA). **(B)** Confusion matrix illustrating the comparison of results between the qPCR assay and IFA. **(C)** Visual appearance of results for 42 clinical samples. The pictures were captured under blue light (470 nm) by a smartphone camera. Bright green fluorescence was observed in positive samples, whereas dim green fluorescence was detected in negative samples.

We further applied this strategy using 42 field-isolated duckling samples exhibiting clinical symptoms such as poor feathering (**Supplementary Figure S3A**) and intestinal embolism (**Supplementary Figure S3B**). Initial screening via IFA classified 20 samples as positive and 22 as negative (**Supplementary Figure S4**). Subsequent diagnostic evaluation using both the RPA-CRISPR/Cas12a and qPCR assays revealed superior performance of the CRISPR-based method. The RPA-CRISPR/Cas12a assay exhibited superior performance, achieving a diagnostic sensitivity of 100% (95% CI: 83.2%–100%) and 95.5% specificity (95% CI: 77.2%–99.9%), with a PPV of 95.2% (95% CI: 76.4%–99.9%) and a NPV of 100% (95% CI: 84.6%–100%) (**Figure 5A**). In contrast, qPCR showed reduced accuracy with a diagnostic sensitivity of 90% (95% CI: 68.4%–98.8%) and 90.9% specificity (95% CI: 73.5%–98.8%), as detailed in the comparative analysis (**Figure 5B**).

To develop an affordable on-site testing platform, we utilized a portable blue light transilluminator for rapid visual readouts. As depicted in **Figure 5C**, positive samples exhibited strong bright green fluorescence under the transilluminator, whereas negative samples showed negligible background fluorescence. This fluorescence-based interpretation exhibited complete concordance with the RPA-CRISPR/Cas12a assay results, validating the transilluminator’s applicability for on-site diagnostics in resource-limited settings.

## Discussion

Infectious diseases among waterfowl have surged in recent years, with significant economic implications. GPV, in particular, has been endemic in various global regions, causing notable economic losses (Chen et al., 2016). Consequently, the development of rapid and accurate diagnostic tools for GPV surveillance is of paramount importance. PCR-based methods are commonly utilized for GPV detection due to their high specificity and sensitivity. Nonetheless, these techniques are constrained to laboratory environments due to their reliance on sophisticated equipment and specialized personnel.

In this study, a visualization method combining RPA with CRISPR/Cas 12a system for rapid, simple, and highly sensitive detection of GPV was developed. The assay provides flexible detection platforms, allowing results analysis through either a real-time fluorescent PCR instrument or a multimode microplate reader. For field applications, a portable blue light transilluminator enables visual inspection, significantly enhancing on-site testing feasibility. Compared to qPCR, the RPA-CRISPR/Cas12a assay demonstrated superior diagnostic accuracy, achieving 100% sensitivity (20/20 true positives) and 95.5% specificity (21/22 true negatives) (**Figure 5A**). In contrast, qPCR assay showed lower sensitivity (90%, with 2/20 false negatives) and specificity (90%, with 2/22 false positives) (**Figure 5B**). This performance advantage was further evident in mock infection experiments, where GPV-DNA was reliably detected at both high (2 dpi) and low (10 dpi) concentrations, while qPCR assay only detected GPV-DNA in blood samples at 2 dpi (**Table 4**). The enhanced sensitivity originates from CRISPR/Cas12a system’s dual-function mechanism: crRNA-guided single-base discrimination ensures precise target recognition, whereas Cas12a’s collateral ssDNase activity enables exponential signal amplification, effectively minimizing nonspecific interference common in primer-dependent methods (Chen et al., 2018).

The assay exhibited exceptional specificity with no cross-reactivity against non-GPV targets (**Figure 4B**), consistent with Cas12a’s sequence-specific recognition. Notably, it achieved a detection limit of 10 copies/*μ*L of for GPV plasmids DNA, outperforming existing nucleic acid amplification methods (**Supplementary Table S1**). For instance, Liu et al. (2019) reported an RPA-vertical flow (VF) assay for GPV detection with a LOD of 2 × 10^2^ copies/μL, which is 20-fold lower than our method. Similarly, Yang et al. (2017) employed LAMP with Eva Green dye to reduce non-specific amplification but achieved only a LOD of 10^2^ copies/*μ*L, requiring higher amplification temperatures (65°C). Through optimized coordination between RPA primers and crRNA, we eliminated aerosol contamination risks associated with RPA-LF assays (Hu F, et al., 2022) and reduced the total detection time to 55 minutes, overcoming the prolonged workflows of qPCR and semi-nested PCR (**Supplementary Table S1**).

Buffer components play a significant role in the spontaneous formation of essential higher-level protein structures (Habimana et al., 2023). Therefore, they may influence the *trans*-cleavage efficiency of Cas12a. While previous studies generally used NEBuffer r2.1 for CRISPR/Cas reactions (Wu et al., 2022; Yang et al., 2022), our findings indicated that the Cas12a protein achieved optimal trans-cleavage efficiency in the Magigen buffer, which contains dithiothreitol (DTT) (see **Supplementary Table S2**) (Chen et al., 2023; Deng et al., 2022; Ding et al., 2018). DTT is crucial for stabilizing enzymes and protecting sulfhydryl groups, thereby enhancing the accuracy of CRISPR nucleic acid detection systems.

To streamline the detection process, employed a rapid nucleic acid lysis strategy, reducing the total assay time to 55 minutes (comprising 5 minutes for nucleic acid release, 15 minutes for RPA, and 35 minutes for CRISPR/Cas12a detection; see **Figure 1**). The use of a portable blue light transilluminator for visual observation underscores the assay’s suitability for on-site GPV detection. Despite the assay’s rapid detection and high specificity, several challenges persist. Primarily, the separation of nucleic acid amplification and CRISPR/Cas12a *trans*-cleavage into two distinct steps introduces complexities. While this two-step approach prevents the loss of the RPA nucleic acid template due to *cis* cleavage by CRISPR/Cas12a and avoids RPA primer degradation from CRISPR/Cas12a trans-cleavage activation (Hu F et al., 2022), it complicates manual handling and increases the risk of cross-contamination during amplicon transfer. To mitigate these risks, several strategies have been proposed, such as separating the isothermal amplification system from the Cas12/crRNA complex (Pang et al., 2020; Wang et al., 2021), utilizing photocleavable linkers (Hu M et al., 2022), or developing a PAM-free one-step assay (Yang et al., 2024). Additionally, quantifying high target concentrations using CRISPR/Cas-based assays remains challenging due to the system’s high sensitivity and limited availability of reporter molecules, which can lead to an early signal plateau (Li et al., 2019). Furthermore, although our assay requires only a portable instrument, there is still a need to develop assays based on lateral flow immunoassays to fulfill the demand for even more rapid diagnostics. Nevertheless, these constraints are anticipated to be addressed in future research endeavors.

## Conclusion

In summary, a novel method integrating RPA with the CRISPR/Cas12a system was successfully established for rapid, visual, and field-deployable detection of GPV. The assay demonstrates outstanding sensitivity and specificity, achieving a rapid turnaround time of only 55 min from samples collection to result generation. Notably, the system eliminates complex instrumentation requirements by incorporating a blue light transilluminator for instant visual interpretation, addressing a critical bottleneck in point-of-care testing. Collectively, this study presents an innovative, on-site approach for GPV detection, holding substantial promise for implementation in resource-limited settings where traditional laboratory infrastructure is lacking.

## Data Availability

The datasets presented in this study can be found in online repositories. The names of the repository/repositories and accession number(s) can be found in the article/**Supplementary Material**.
