# Supplementary material for: A combination of recombinase polymerase amplification with CRISPR technology rapidly detects goose parvovirus with high accuracy and sensitivity
Source: Front Cell Infect Microbiol. 2025 Jun 16;15:1566603. doi: 10.3389/fcimb.2025.1566603 (PMC12206813; doi:10.3389/fcimb.2025.1566603)
Supplement: Supplementary file 1 [file DataSheet1.docx]

**Supporting Information**

**A combination of recombinase polymerase amplification with CRISPR technology rapidly detects goose parvovirus with high accuracy and sensitivity**

Xiuqin Chen^1^, Shizhong Zhang^1^, Su Lin^1^, Shao Wang^1^, Meiqing Huang^1^, Shaoying Chen^1^, Shilong Chen^1*^

^1^ Institute of Animal Husbandry and Veterinary Medicine, Fujian Academy of Agricultural Science, Fuzhou, Fujian 350013, China；

*****Corresponding author: Shilong Chen


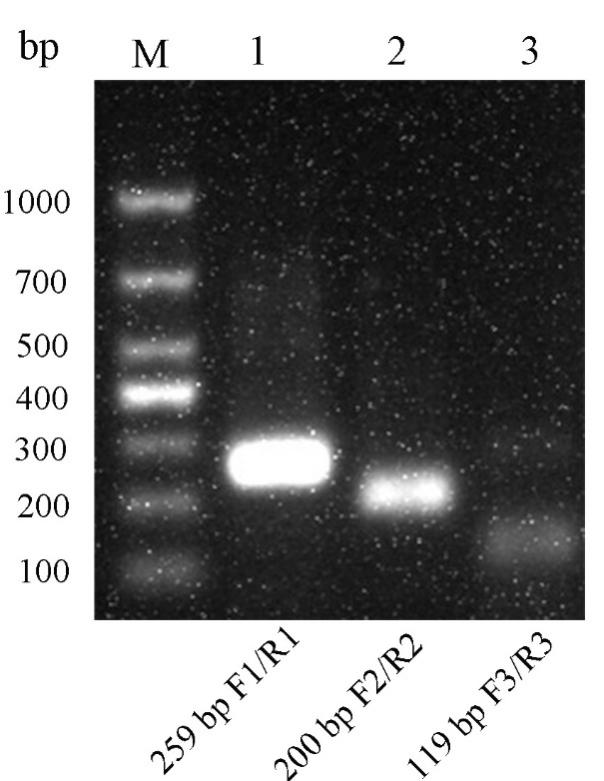


**Figure S1** The optimal RPA primer pairs for screening GPV. Optimization was performed using agarose gel electrophoresis (2%). For the template, 10^5^ copies/*μ*L of standard plasmids were used. Lane M: DL1000 Marker, lane “1-3” represents the first, second, and third pairs of primers, respectively. The expected sizes of the RPA products were 259 bp, 200 bp, and 119 bp. The sizes of the amplified products were consistent with the expectations (Table 1).


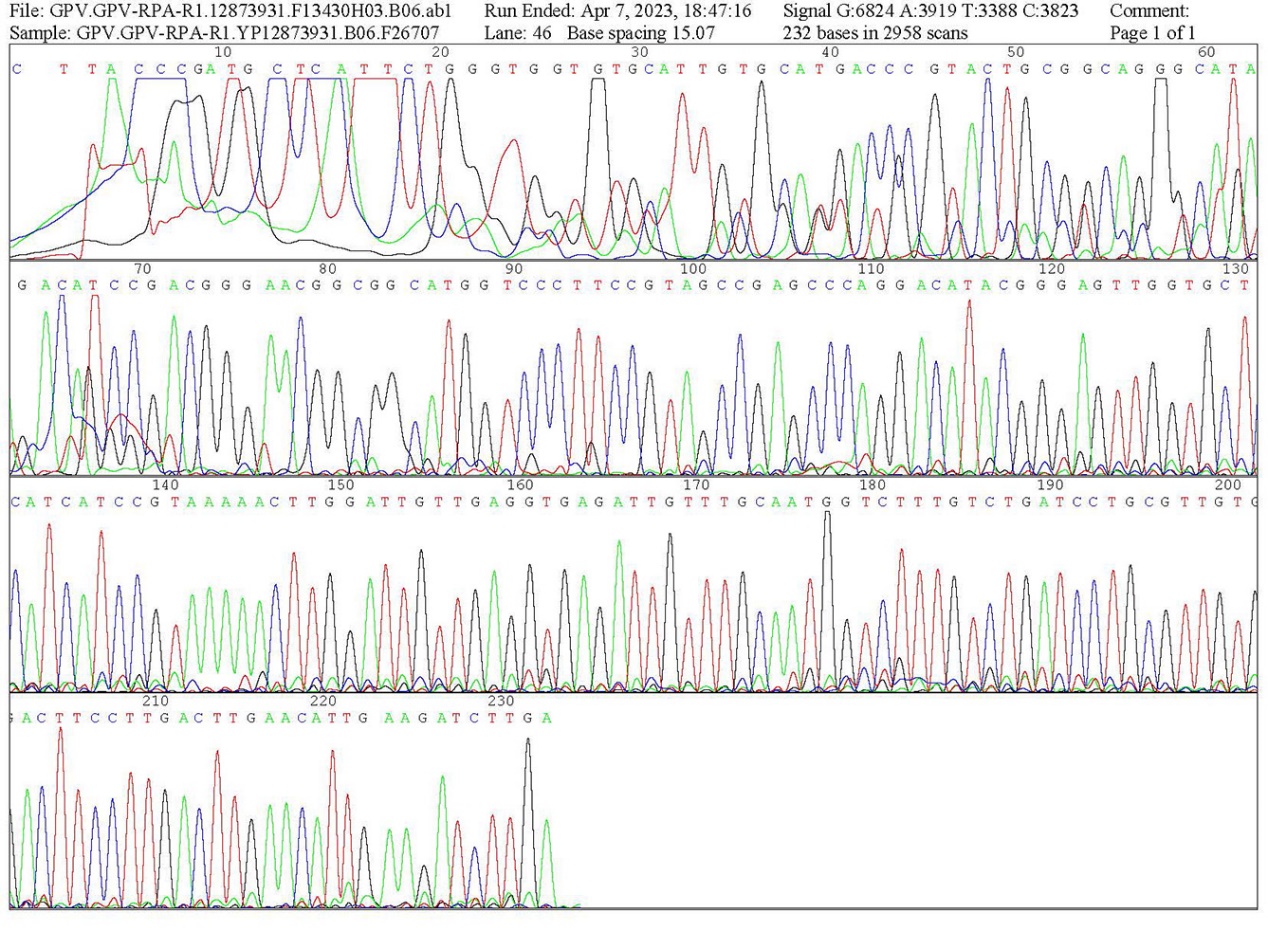


**Figure S2** Sanger sequencing result of the RPA-amplified products of the first pair of primers targeting the *VP3* gene.

Notes: The RPA amplification sequence of the *VP3* gene is listed as follows:

>GPV *VP3*

TCAAGATCTTCAATGTTCAAGTCAAGGAAGTCACAACGCAGGATCAGACAAAGACCATTGCAAACAATCTCACCTCAACAATCCAAGTTTTTACGGATGATGAGCACCAACTCCCGTATGTCCTGGGCTCGGCTACGGAAGGGACCATGCCGCCGTTCCCGTCGGATGTCTATGCCCTGCCGCAGTACGGGTACTGCACAATGCACACCAACCAGAATGGAGCACGGTTCAATGACCGTAGCGCATTCTACTGCTTAGAGA


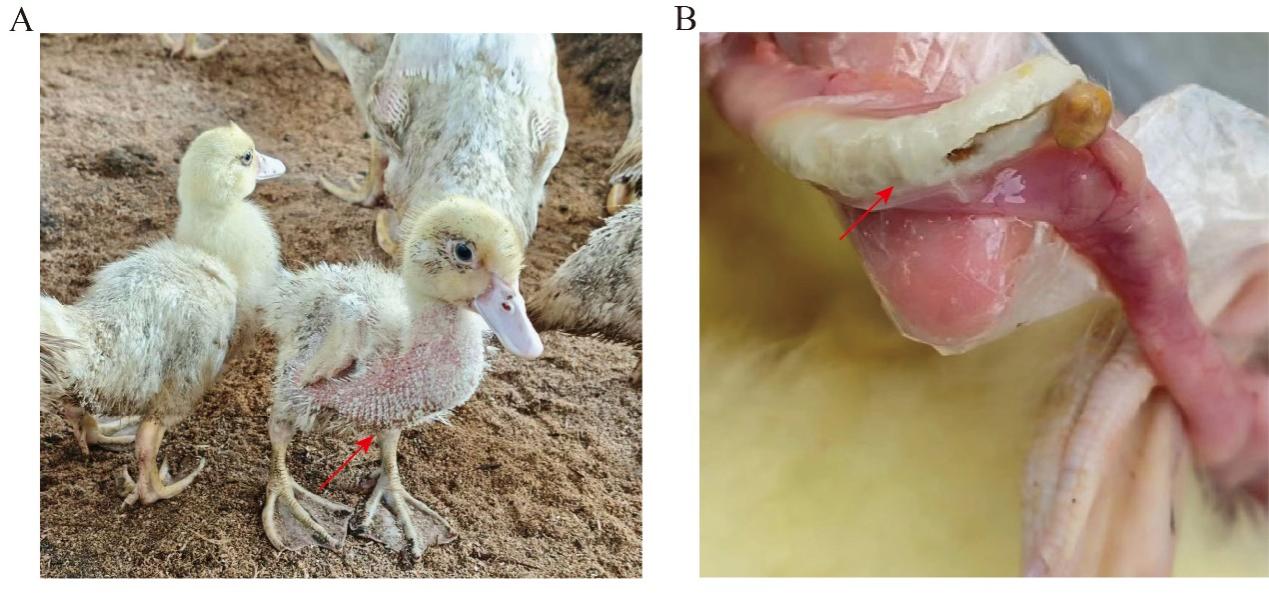


**Figure S3** Clinical symptoms and pathological changes observed in diseased ducks collected from the clinical breeding farm. Diseased ducks present dysplastic feathers (A), and intestinal embolism (B) evident upon gross necropsy.


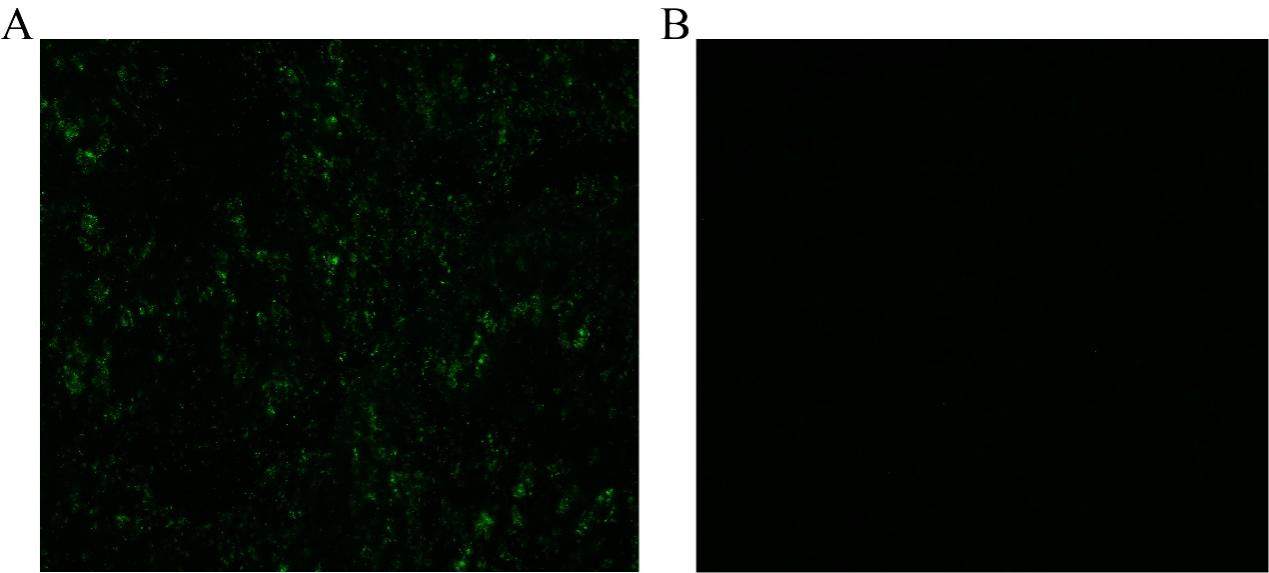


**Figure S4** Detection of GPV antigen by indirect immunofluorescence assay (×40). Utilizing MAb E16, which is specific to GPV, as the primary detection antibody, the secondary antibody chosen was FITC-conjugated goat anti-mouse immunoglobulin. (A) Positive clinical samples; (B) Negative clinical samples.

**Table S1**: Comparison of different detection methods for GPV nucleic acid.

| Analytical method | Sensitivity | Temperature | Detection time ^e^ | Visualization | POCT ^f^ | Ref. |
| --- | --- | --- | --- | --- | --- | --- |
| Semi-nested PCR | 10^2^  copies/*μ*L | Temperature cycling | > 60 min | No | No | ^[1]^ |
| TaqMan based RT-PCR ^a^ | 10^2^  copies/*μ*L | Temperature cycling | > 60 min | No | No | ^[2]^ |
| SYBR Green II RT-PCR | 1.96 × 10^1^  copies/*μ*L | Temperature cycling | > 60 min | No | No | ^[3]^ |
| RPA-VF ^b^ | 2 × 10^2^  copies/*μ*L | A constant temperature of 37℃ | 5-10 min | Yes | Yes | ^[4]^ |
| qLAMP ^c^ | 1 × 10^2^  copies/*μ*L | A constant temperature of 65℃ | 60 min | Yes | Yes | ^[5]^ |
| RPA-*Pf*Ago ^d^ | 3.89 × 10^2^ copies/*μ*L | A constant temperature of 36℃ to 39℃ | 50 min | Yes | Yes | ^[6]^ |
| RPA-CRISPR/Cas12a | 10^1^  copies/*μ*L | A constant temperature of 37 ℃ | 50 min | Yes | Yes | This work |

^a^ RT-PCR: real-time PCR

^b^ RPA-VF: recombinase polymerase amplification was combined with a vertical flow visualization strip

^c^ qLAMP: quantitative loop-mediated isothermal amplification

^d^ *Pf*Ago: *Pyrococcus furiosus* argonaute

^e^ Exclude the time of nucleic acid extraction

^f^ POCT: point-of-care testing

**Table S2**: The components of four commercial reaction buffers.

| Components | Magigen buffer | NEBuffer r2.1 | NEBuffer r3.1 | CutSmart buffer |
| --- | --- | --- | --- | --- |
| NaCl | 500 mM | 50 mM | 100 mM | - |
| Tris-tricine | 100 mM | **-** | **-** | - |
| MgCl_2_ | 100 mM | 10 mM | 10 mM | - |
| DTT ^a^ | 10 mM | **-** | **-** | - |
| Tris-HCl | **-** | 10 mM | 50 mM | - |
| BSA ^b^ | **-** | 100 µg/mL | 100 µg/mL | - |
| Potassium Acetate | - | - | - | 50 mM |
| Tris-acetate | - | - | - | 20 mM |
| Magnesium Acetate | - | - | - | 10 mM |
| Recombinant Albumin | - | - | - | 100 µg/mL |

^a^ DTT, dithiothreitol

^b^ BSA, bovine serum albumin

**Supplementary References**

[1] LI PENGFEI, ZHANG RUIHUA, CHEN JUNHAO, et al. Development of a duplex semi-nested PCR assay for detection of classical goose parvovirus and novel goose parvovirus-related virus in sick or dead ducks with short beak and dwarfism syndrome[J]. Journal of Virological Methods, 2017,249:165-169.

[2] WANG JIANCHANG, WANG JINFENG, CUI YUAN, et al. Development of a taqman-based real-time PCR assay for the rapid and specific detection of novel duck-origin goose parvovirus[J]. Molucular and Cellular Probes, 2017,34:56-58.

[3] LUO QIHUI, CHEN BING, XU JING, et al. Development of a SYBR Green II real-time polymerase chain reaction for the clinical detection of the duck-origin goose parvovirus in China[J]. Intervirology, 2018,61(5):230-236.

[4] LIU WEN-JUN, YANG YOU-TIAN, DU SI-MIN, et al. Rapid and sensitive detection of goose parvovirus and duck-origin novel goose parvovirus by recombinase polymerase amplification combined with a vertical flow visualization strip[J]. Journal of Virological Methods, 2019,266:34-40.

[5] YANG JING, CHEN HAO, WANG ZHENZHONG, et al. Development of a quantitative loop-mediated isothermal amplification assay for the rapid detection of novel goose parvovirus[J]. Frontiers in Microbiology, 2017, 8:2472.

[6] LIU YAQUN, CHEN LIANGHUI, ZHANG ZHENXIA, et al. Development and application of a novel recombinase polymerase amplification-Pyrococcus furiosus argonaute system for rapid detection of goose parvovirus[J]. Poultry Science, 2024,103(10):104141.
